# Effect of time and depth of insemination on fertility of Bharat Merino sheep inseminated trans-cervical with frozen-thawed semen

**DOI:** 10.1186/2055-0391-56-8

**Published:** 2014-07-24

**Authors:** Davendra Kumar, Syed Mohammed Khursheed Naqvi

**Affiliations:** Division of Animal Physiology and Biochemistry, Central Sheep and Wool Research Institute, Avikanagar, 304501 via Jaipur, Rajasthan India

**Keywords:** Frozen semen, Ewe, Non-invasive intrauterine insemination, Lambing

## Abstract

**Background:**

Artificial insemination (AI) can serve as a powerful tool to the sheep owners for making rapid genetic progress of their flock. The AI in sheep is mostly performed using fresh semen with two reasons i) lambing rate following trans-cervical AI with frozen semen is limited by the inability of frozen-thawed sperm to transit the cervix and ii) the need of circumventing the cervical barrier through laparoscope aided intrauterine AI. Therefore, AI with frozen-thawed semen is not as widespread in sheep as it is in other domestic species. However, to get maximum benefits through the use of AI, frozen-thawed semen is a prerequisite because instead of high fertility, the short shelf life of fresh semen coupled with a limitation on the number of insemination doses achievable per unit time restricts the widespread use of individual sires. Therefore, in order to enhance lambing rate, a total of 240 trans-cervical artificial inseminations with frozen-thawed semen were performed in Bharat Merino ewes during autumn season either once in the evening (G-I, 10 h after onset of estrus, n = 100) or twice (G-II, 14 h and 22 h after onset of estrus, n = 140) i.e. once in the morning and again in the evening.

**Results:**

The pregnancy rate (proportion of pregnant ewes confirmed by ultrasonography at day 40) and lambing rate (proportion of ewes lambed) were higher in G-II as compared to G-I (26.4 vs 20% and 19.3 vs 10%, respectively). The difference in lambing rates was statistically (P < 0.05) significant. The depth of insemination within cervico-uterine tract had no significant effect on pregnancy and lambing rates.

**Conclusions:**

The results indicate that lambing rate in sheep following TCAI with frozen-thawed semen was significantly influenced by time of inseminations. Two inseminations after 14 and 22 h of onset of estrus enhanced the lambing rates of Bharat Merino sheep as compare to single insemination after 10 h of onset of estrus. The TCAI technique with frozen-thawed ram semen is promising and may serve as a valuable tool for genetic improvement of sheep breeds. Research efforts are going on worldwide to overcome the poor fertility following TCAI with frozen-thawed semen.

## Background

Artificial insemination (AI) can serve as a powerful tool to the sheep owners for making rapid genetic progress of their flock. The AI is expected to play a crucial role for large-scale production and multiplication of fecundity gene carrier germplasm via introgression of fecundity gene into the non-prolific sheep breeds. The AI in sheep is mostly performed using fresh semen, since fertility following AI with frozen semen is limited by the inability of frozen-thawed sperm to transit the cervix. Even when very large number of motile spermatozoa is deposited in the cervix, fertility is lower for frozen semen than for fresh semen [1]. Therefore, AI with frozen-thawed semen is not as widespread in sheep as it is in other domestic species. However, to get maximum benefits through the use of AI, frozen-thawed semen is a prerequisite because instead of acceptable fertility, the short shelf life of fresh semen coupled with a limitation on the number of insemination doses achievable per unit time restricts the widespread use of individual sires.

In all domestic species AI with frozen-thawed semen gives acceptable pregnancy rates only when semen is deposited intrauterine. In sheep, the cervix is a formidable barrier to penetration for trans-cervical intrauterine deposition of frozen-thawed semen due to its caudally facing, eccentric series of four to five funnel-like rings [2–4]. At present satisfactory and reliable lambing results with frozen-thawed semen are only obtainable by circumventing the cervical barrier through laparoscope aided intrauterine AI [5–7]. But the cost, invasive nature and need for professional labor limits its wider use [8]. The development of trans-cervical artificial insemination (TCAI) technique has got immense potential for using frozen-thawed semen because of its non-invasive nature and as an alternative technique for invasive laparoscope-aided intrauterine AI [9]. The pregnancy and lambing rates achieved through TCAI with frozen semen is not still acceptable and there is need to improve lambing rates following TCAI. Besides breed, frozen-thawed semen quality, sperm numbers, ewe condition and time of insemination, the frequency of insemination to meet out the availability of sperm near ovulation for successful fertilization, and catheter penetration (site of semen deposition) are important factors which may influence success rate of TCAI.

The time of insemination is related to the time of ovulation. Ovulation occurs around the end of estrus. Insemination should be performed at a time sufficiently before ovulation so that by the time of ovulation a large population of sperm is established in the ampulla, the site of fertilization. In general, with twice daily inspections for heat detection, if animal showing estrus in the morning it should be inseminated in the evening (10 h after onset of estrus), and if animal showing estrus in the evening it should be inseminated in the morning (14 h after onset of estrus). However, time of insemination needs to be adjusted according to whether the semen is fresh or frozen‑thawed, since the former should have a fertilizing life in the female reproductive tract well in excess of 24 h, while for the latter the fertilizing life may not be more than 12 h. Therefore, with frozen-thawed semen two inseminations 8 h apart may play role in providing large population of live sperm (owing to the short life span of frozen-thawed sperm) at the site of fertilization by the time of ovulation. Keeping this in view a fertility trial was conducted in Bharat Merino ewes to determine the influence of time of insemination (10 h Vs 14 & 22 h after onset of estrus) and depth of insemination within cervico-uterine tract on pregnancy and lambing rates following TCAI with frozen-thawed ram semen.

## Results and discussion

The results are presented in Table 1. The time of insemination had significant (p < 0.05) effect on lambing rates with higher values in G-II as compare to G-I. However, time of insemination had no significant effect on pregnancy rates. The depth of insemination within cervico-uterine tract i.e. cervical penetration had no significant effect on pregnancy and lambing rates. However, both pregnancy and lambing rates were slightly higher in case of full cervical penetration of insemination needle.

**Table 1:** **Effect of timing and depth of insemination within cervico-uterine tract on pregnancy and lambing rates of Bharat Merino ewes inseminated with frozen-thawed semen**

Timing of insemination after onset of estrus	Depth of insemination within cervico-uterine tract									Overall		
	Full (uterine)			Mid (3 ^rd^to last cervical fold)			Partial (os to 2 ^nd^cervical fold)					
	n	Pregnancy rate (%)	Lambing rate (%)	n	Pregnancy rate (%)	Lambing rate (%)	n	Pregnancy rate (%)	Lambing rate (%)	n	Pregnancy rate (%)	Lambing rate (%)
**14 & 22 h**	89	31.5	20.2	23	17.4	17.4	28	17.9	17.9	**140**	**26.4**	**19.3** ^**a**^
**10 h**	73	21.9	11.0	12	16.7	8.3	15	13.3	6.7	**100**	**20**	**10** ^**b**^
**Overall**	**162**	**27.2**	**16.0**	**35**	**17.1**	**14.3**	**43**	**16.3**	**14.0**	**240**	**23.3**	**15.4**

Values with different superscripts in same column are significantly (P < 0.05) different.

The success rate attained in this study in terms of pregnancy and lambing rates following TCAI of ewes with frozen-thawed semen for one oestrus cycle is at par with other reports [10–16]. The variation in the results may be due to the differences in breed, season, feeding, management, freezing and thawing procedures, insemination dose (number of spermatozoa per straw), equipment used for TCAI, level of penetration of catheter into the cervix, time and frequency of insemination in relation to ovulation and onset of estrus whether natural or synchronized. Moreover, the process of manipulating an insemination catheter through the cervix has been linked to reductions in pregnancy and lambing rates. It has been suggested that cervical trauma and vaginal/cervical stimulation caused by the catheterization may activate pathways that are associated with uterine immune defenses and that interrupt pregnancy at its early stages [17, 18, 14]. It is believed that frozen-thawed sperm are less motile and lack stamina to transverse the highly viscous cervical mucus, because of ultrastructural, biochemical and functional changes undergone by a large sperm population but phagocytosis of the sperm by leukocytes is also considered a cause of the reduced fertility [19]. Ultrastructural changes mainly affect sperm membranes because during the frozen-thawed process there is a redistribution of lipids that alters lipid-lipid and lipid-protein relations, which are necessary for the normal function of sperm membranes [20].

The higher lambing rate of the ewes inseminated 14 and 22 h after onset of estrus as compare to ewes inseminated 10 h after onset of estrus indicates that the time and/or frequency of insemination of frozen-thawed semen is a critical determinant of fertility, due primarily to the significantly reduced lifespan of frozen-thawed semen in the female reproductive tract. However, Salamon [21] reported no difference between the mean lambing results for frequency of inseminations i.e. single (44.4%, 59/133) and double insemination (46.2%, 61/132) with frozen semen. The effect of double insemination depends upon the time when the first insemination is performed in relation to ovulation. So, the effect of the double insemination is more reliable when the first insemination is carried out at the beginning of estrus than when it takes place in the middle or at the end of estrus. The beneficial effect of two insemination at 14 and 22 h after onset of estrus in the present study may be because insemination is artificial, natural sexual behavior is absent and the male and female reproductive events have to be carefully synchronized to minimize the gamete waiting time before fertilization occurs. Moreover, two insemination may prevent from the possibility that insemination is practiced too early in relation with ovulation as seems in the G-I where insemination was done at 10 h after onset of estrus.

In the present study the depth of cervical penetration i.e. site of semen deposition did not influence statistically (p < 0.05) pregnancy and lambing rates, however, these are slightly more in case of uterine deposition as compared with cervical deposition. Taking into consideration the anatomy of the sheep cervix, a highly complex fibrous structure with many folds that obliterate the lumen, it is understandable. In partial cervical penetration, a part of semen or the whole quantity can flow back into the vagina where the acidic pH is unfavorable condition for the sperm. In mid cervical penetration, a large number of sperm practically come to a dead end in the rings of the cervix and cannot get into the body of the uterus. In full cervical penetration, semen enters directly into the body of the uterus. The depth of cervical penetration is affected by the anatomy of cervical lumen. Cervices with a less convoluted lumen are more penetrable [22]. The eccentric folds are the ones most difficult to pass and they influence the design of the tip of the AI needle [23]. Generally, the second fold is more misaligned than the first and third, which originates a narrow cervical lumen [3, 24]. The depth from the os to the first 2 folds of the cervix has been shown to be the major factor limiting the cervical penetrability [25, 26]. Several authors have reported that low fertility obtained when semen is applied via the cervix, is associated with low penetration. The high fertility rate on intrauterine deposition of semen has also been reported by several workers [10, 12, 27–30]. However, recently it has also been reported that the degree of catheter penetration into the cervix had no correlation with fertility of ewes inseminated with frozen-thawed semen [16, 31].

The linear‒array real‒time ultrasound scanning by the transabdominal route is a reliable method for (early) pregnancy diagnosis in sheep with accuracy of more than 90% [32, 33]. The difference in pregnancy and lambing rates in the present study may be due to fetal mortality or unobserved abortions that took place after day 40 of pregnancy. Dixon *et al.* [34] reported that approximately 19.9% of the ewes experienced late embryonic loss, fetal loss, or both; and 21.2% of the embryos or fetuses were lost from d 25 to term. The proportions of fetuses lost were associated with breed type, concentrations of progesterone, estradiol and vascular endothelial growth factor. Cryoinjuries of spermatozoa associated with freezing and thawing, and manipulations associated with TCAI technique can all contribute to fetal mortality, probably due to inadequate vascular growth in the endometrium and perhaps conceptus. Langford *et al.* [35] and Olesen [36] also reported increased embryonic mortality between day 18 and term when frozen‒thawed semen was used. The contribution of false positive results to the differences between pregnancy and lambing rates may not be ignored.

## Conclusions

The results of this study indicate that lambing rate in sheep following TCAI with frozen-thawed semen was significantly influenced by time of inseminations. Two inseminations after 14 and 22 h of onset of estrus enhanced the lambing rates of Bharat Merino sheep as compared to single insemination after 10 h of onset of estrus. The TCAI technique with frozen-thawed ram semen is promising and may serve as a valuable tool for genetic improvement of sheep breeds. Research efforts are going on worldwide to overcome the poor fertility following TCAI with frozen-thawed semen. Improvement in the freezing protocol and the use of exogenous cervical dilatators in sheep have been investigated but a much better understanding of sheep cervical physiology and the mechanism of natural cervical dilatation at estrus is required to facilitate TCAI for sheep with frozen-thawed semen to achieve acceptable fertility.

## Methods

The study was conducted with the approval of Institute Animal Ethics Committee (IAEC), which is in the semi-arid tropical area of the India at 75°-28′E longitude, 26°-26′N latitude and at an altitude of 320 m above mean sea level. The climate of this place is typically hot with yearly minimum and maximum temperature of 4° and 46°C, respectively. The annual rainfall ranges from 400 to 700 mm with an erratic distribution throughout the year.

**Figure 1:**
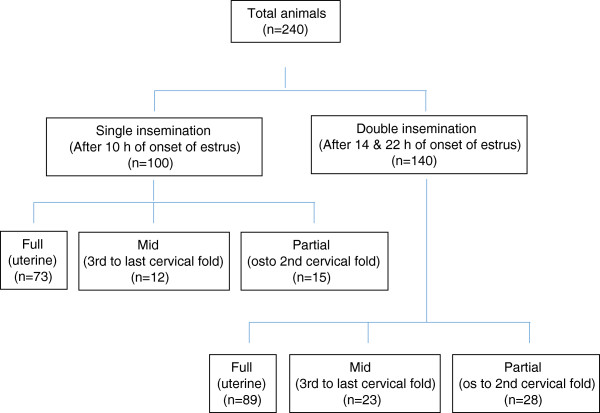
**Experimental design.**

A total of 240 TCAI with frozen-thawed semen were performed in 2–4 year old Bharat Merino ewes having body weight of 35–42 kg, during autumn season when major breeding activities commence at the institute farm. Bharat Merino sheep has been evolved at Central Sheep and Wool Research Institute, Avikanagar to cater the indigenous demand of fine wool for woollen sector by crossbreeding native ewes (Nali, Chokla, Malpura and Jaisalmeri) with exotic Rambouillet/Soviet Merino rams and stabilizing the population at 75% exotic inheritance [37]. Twelve adult Bharat Merino rams of 3–4 years of age and 60–75 kg body weights were used as semen donors. The rams and ewes were maintained under the semi-intensive management system before and after inseminations. All the animals were allowed for 8–10 h daily grazing on natural vegetation interspersed with seasonal shrubs, grasses and forbs (*Achyranthes aspera, Commelina forskalaei, Eleusine aegypticae and Sorghum helepense)*. In addition to grazing, they were provided a concentrate mixture of 300 g/day. After grazing, the animals were housed in a chain-link fence enclosure having asbestos sheet roof open from all the sides (Figure 1).

The semen of individual rams was frozen well in advance and kept cryopreserved till the day of insemination. On the day of freezing, ejaculates were obtained from donor rams in quick succession by artificial vagina after mounting on the restrained estrus ewe secured in the service crate. The semen samples were evaluated for volume, consistency, wave motion (0–5 scale), concentration (photometrically) and percentage of motile spermatozoa (0-100%). Ejaculates having thick consistency, rapid wave motion, 90% initial motility and more than 3 × 10^9^ spermatozoa per ml were immediately diluted with a TEST-yolk-glycerol extender [38] at 25°C to a final concentration of 1 × 10^9^ spermatozoa per ml. The extended semen samples from individual ram were frozen in a Planer R-204 programmable cell freezer under controlled cooling and freezing rates [39]. Briefly, diluted samples were aspirated into 0.5 ml size French plastic straws (IMV Technologies, L’ Aigle, France), sealed with polyvinyl alcohol powder, submerged in water kept in a rectangular glass tray at 25°C and after drying straws were loaded vertically in the programmable cell freezer pre-cooled to 25°C. Controlled-rate cooling of straws was initiated in the cell freezer at the linear rate of 0.15°C per min from 25 ° to 5°C followed by a holding time of 2 h at 5°C and freezing from 5° to -125°C at the rate of 25°C per min and then plunged into liquid nitrogen for storage until required. Thawing of individual straws was done at 60°C for 10 second in a water bath just prior to TCAI. Post-thaw attributes of spermatozoa were objectively assessed by a computer-assisted semen analysis technique (Hamilton-Thorn HTM-S version 7.2 Y motility analyzer) straw per straw. Single straw (0.25 ml French straw having 250 million sperm) of frozen-thawed semen with more than 50% post-thaw motility was immediately used for TCAI of one ewe.

Estrus was detected in ewes twice daily (07:00 and 19:00) by parading adult rams, fitted with an apron. Ewes were inseminated for one cycle either once, only in the morning (G-I, single insemination, n = 100) or twice, once in the morning and again in the evening (G-II, double inseminations, n = 140). Ewes exhibiting estrus in the morning along with ewes in estrus on the previous evening were brought daily to the semenology laboratory for insemination. Ewes exhibited estrus on previous evening were kept in G-II and inseminated twice 8 h apart i.e. between 09:00 to 10:00 as well as 17:00 to 18:00 (14 h and 22 h after onset of estrus). Ewes exhibited estrus same day in the morning were kept in G-I and inseminated once between 17:00 to 18:00 (10 h after onset of estrus). The inseminations were made using individual ram semen allotted to the particular ewe as per the breeding plan (improvement by selective breeding) of Bharat Merino sheep.

The equipment and cradle used for TCAI were fabricated as described earlier by Naqvi *et al.* [12]. The ewes were restrained in the cradle in dorsal decumbency with the hind quarter elevated so that the vulva was positioned at an angle of 80 to 90° from the ground. The insemination was performed throughout the study by the same inseminator using the procedure of Naqvi *et al.* [12]. In brief, the speculum with plunger lubricated with small amount of medical gel was introduced into the vagina and external os was located after removal of the plunger with the aid of the light. The needle was gently introduced into cervix and manipulated through the cervical folds into the deepest location possible. A maximum time limit of 5 minutes was set to achieve full penetration of the cervix. If full penetration had not been achieved semen was deposited in the cervix. The depth of cervical penetration was scored as: (i) os to second fold: partial, (ii) third to last fold: mid and (iii) needle inserted to its full length: full (uterine deposition).

The inseminated ewes were closely observed for return in estrus over two-cycle period and until lambing. At 40 days of gestation, all the inseminated ewes were examined for the presence of fetuses by using a real time Desktop Veterinary Ultrasound Scanner system equipped with convex array 5.0 MHz/40R/60D transducer (Model SA-600 V, MEDISON Company, Limited. Korea). Data on pregnancy rate (proportion of pregnant ewes confirmed by ultrasonography) and lambing rate (proportion of ewes lambed) were recorded. The data were statistically analyzed by chi square test as per Snedecor and Cochran [40] to determine the effect of time of inseminations and depth of insemination within cervico-uterine tract on pregnancy and lambing rates.
